# Sources of Medical Information for Oncology Physicians During the COVID-19 Pandemic: Results From a National Cross-Sectional Survey

**DOI:** 10.1093/jncics/pkaa095

**Published:** 2020-10-24

**Authors:** Helen M Parsons, Rachel I Vogel, Anne H Blaes, Emil Lou, Heather Beckwith, Jianling Yuan, Jane Yuet Ching Hui

**Affiliations:** 1 Division of Health Policy and Management, School of Public Health, University of Minnesota, Minneapolis, MN, USA; 2 Department of Obstetrics, Gynecology and Women’s Health, University of Minnesota, Minneapolis, MN, USA; 3 Division of Hematology, Oncology, and Transplantation, University of Minnesota, Minneapolis, MN, USA; 4 Department of Radiation Oncology, University of Minnesota, Minneapolis, MN, USA; 5 Department of Surgery, University of Minnesota, Minneapolis, MN, USA

## Abstract

Because the coronavirus disease 2019 (COVID-19) has completely transformed the accepted norms and approaches to cancer care delivery in the United States, we sought to understand the sources of medical information that oncology physicians seek and trust. We recruited 486 oncology physicians to an anonymous cross-sectional online survey through social media from March 27, 2020, to April 10, 2020, with 79.2% reporting their sources of medical information during the COVID-19 pandemic. We found a diverse array of reported sources for COVID-19 information that most commonly included professional societies (90.7%), hospital or institutional communications (88.6%), and the Centers for Disease Control and Prevention (69.9%); however, trust in these sources of information varied widely, with professional societies being the most trusted source. These results highlight the important role that professional societies, hospitals, and the Centers for Disease Control and Prevention play in ensuring dissemination of consistent, high-quality practice recommendations for oncology physicians.

The coronavirus disease 2019 (COVID-19) has completely transformed the accepted norms and approaches to cancer care delivery in the United States (1). To stem growth of the pandemic and protect immunocompromised patients, governments, health systems, and professional societies have upended their treatment guidelines to include widespread telemedicine, postponing or cancelling nonessential procedures, and modifying courses of treatment (2-4). Although many overarching recommendations are consistent across organizations, the rapid spread of COVID-19 has resulted in continually evolving guidance on treatment and protective measures for patients and physicians. Therefore, understanding the sources of medical information that oncology physicians seek and trust is critical to ensure consistent recommendations and practice concordant with the most current knowledge.

We enrolled 486 eligible oncology physicians (surgeons, medical and radiation oncologists) onto an anonymous, cross-sectional, online survey using snowball convenience sampling methods over social media platforms (Facebook, LinkedIn, Twitter, American Cancer Society discussion forums) (5) from March 27, 2020, to April 10, 2020. All participants were provided with information about the study and confirmed eligibility as an oncology physician before completing the survey. Eligibility criteria included being a physician (MD or DO) who treats cancer patients in the United States, age 18 years and older, and ability to read and write in English. Survey items included physician demographics, practice characteristics, cancer treatment decisions, and sources of medical information. Validated measures were used or modified as appropriate (Supplementary Materials, available online) and usability and technical functionality was tested before fielding the questionnaire. Participants could review and change answers as they progressed through the survey. Additional details on survey design can be found elsewhere (5). A total of 385 physicians (79.2%) reported their sources of medical information during the COVID-19 pandemic and degree of trust in these sources for general health information. Survey data were collected and stored using REDCap (6). We analyzed frequencies and conducted χ^2^ tests of the relationships between sources of COVID-19 information and trust in these sources by physician characteristics using SAS 9.4 (Cary, NC). *P* less than .05 was considered statistically significant. Statistical tests were 2-sided. The University of Minnesota Institutional Review Board approved the study.

In our study, 56.1% of participants were surgeons, 23.6% medical oncologists, 14.9% radiation oncologists, and 4.9% other oncology physicians (Table 1). Participants were more commonly female (63.0%), with an average age of 45.9 years (SD = 9.7 years). The majority practiced in larger hospitals with 500 and more beds (46.0%), were affiliated with academic institutions (54.3%), and treated a wide range of cancers. Participants reported a variety of sources for COVID-19 information, most commonly professional society recommendations or guidelines (90.7%), hospital or institution communications (88.6%), and the Centers for Disease Control and Prevention (CDC; 69.9%) (Table 2). However, 60.3% also derived COVID-19 information from social media (physician groups) and traditional news or media (57.7%). Physician trust in information about health and medical topics varied widely by source. Among all physicians, 63.1% reported trusting information from professional society recommendations or guidelines “a lot,” followed by literature searches (50.2%), the World Health Organization (46.4%), and the CDC (45.3%). Few reported “a lot” of trust in social media (0.5%) or news or media reports (1.8%), although over one-quarter (25.2%) showed confidence in physician-only social media groups. When searching for COVID-19 information, over 73.0% were concerned about the quality of information, 42.1% felt it took a lot of effort to get needed information, and 42.1% were frustrated during their search; however, only 16.6% felt the information was hard to understand. Although sources of information generally did not vary by physician characteristics, we found surgeons were more likely to report using society recommendations (96.7% vs 78.0% medical oncologists and 84.2% radiation oncologists; *P* < .001) and were less likely to use grand rounds (31.0% vs 51.6% medical oncologists and 47.4% radiation oncologists; *P* < .001; data not shown).

**Table 1.** Demographic and clinical practice characteristics of oncology physicians (N = 385)

Characteristic	No. (%)
Mean age, y (SD) (n = 342)	45.9 (9.7)
Mean years in practice (SD) (n = 363)	13.0 (10.1)
Sex	
Male	137 (35.5)
Female	241 (63.0)
Nonbinary gender identification	4 (1.0)
Missing	3 (0.5)
Race	
White, non-Hispanic	276 (71.7)
Asian Indian	38 (9.9)
Chinese	17 (4.4)
Hispanic	12 (3.1)
Other	25 (6.5)
Missing	17 (4.4)
Medical specialty	
Surgeon	216 (56.1)
Medical oncology	91 (23.6)
Radiation oncology	57 (14.9)
Other	19 (4.9)
Missing	2 (0.5)
Practice at an academic institution	
No	172 (44.7)
Yes	209 (54.3)
Missing	4 (1.0)
Hospital size	
Small hospital (<100 beds)	30 (7.9)
Medium hospital (100-499 beds)	163 (42.4)
Large hospital (≥500 beds)	177 (46.0)
Ambulatory clinic only (no inpatients)	12 (3.2)
Missing	3 (0.5)
Type of community (practice)	
Rural area	18 (4.7)
Small city or town	82 (21.3)
Suburb near a large city	95 (24.7)
Large city	182 (47.3)
Missing	8 (2.0)
Cancers treated (choose all that apply)^a^	
GU (bladder, renal, prostate)	68 (17.6)
Bone	37 (9.6)
Breast	221 (57.4)
Gynecologic	68 (17.7)
Colorectal	161 (41.8)
Head or neck	85 (22.0)
Hematologic malignancy	88 (22.8)
HPB (liver, pancreatic)	115 (29.9)
Lung	74 (19.2)
Skin or soft tissue	161 (41.8)
Other	72 (18.7)
Missing	8 (2.1)
COVID-19 cases in state where practicing (as of April 3, 2020^b^), No.	
101-500	13 (3.4)
501-1000	80 (20.8)
1001-5000	136 (35.3)
5001 or more	135 (35.1)
Missing (did not provide state where practice)	21 (5.4)

a Percentages do not sum to 100. COVID-19 = coronavirus disease 2019; GU = genitourinary; HPB = Hepato-Pancreatico-Biliary.

b The number of COVID-19 cases in each state based on data reported to the Centers of Disease Control and Prevention (7).

**Table 2.** Sources of COVID-19 information for oncology physicians

Question	No. (%)
Total sample	385 (100)
Which of the following sources do you use for information about COVID-19? Select all that apply^a^	
Physician grand rounds or talks	158 (41.0)
Hospital or institution communications or emails	341 (88.6)
Social media	118 (30.7)
Social media: physician-only groups	232 (60.3)
Literature search	173 (44.9)
News or media reports	222 (57.7)
Professional society recommendations or guidelines	349 (90.7)
CDC reports	269 (69.9)
World Health Organization reports	166 (43.1)
Other	10 (2.6)
In general, how much would you trust information about health or medical topics from…	
Physician grand rounds or talks	
Not at all	9 (2.3)
A little	26 (6.8)
Some	147 (38.1)
A lot	192 (49.9)
Missing	11 (2.9)
Hospital or institution communications or emails	
Not at all	13 (3.4)
A little	38 (9.9)
Some	194 (50.4)
A lot	135 (35.1)
Missing	5 (1.2)
Social media	
Not at all	139 (36.2)
A little	150 (38.9)
Some	82 (21.3)
A lot	2 (0.5)
Missing	12 (3.1)
Social media: physician-only groups	
Not at all	18 (4.6)
A little	88 (22.9)
Some	174 (45.2)
A lot	97 (25.2)
Missing	8 (2.1)
Literature search	
Not at all	4 (1.0)
A little	24 (6.2)
Some	153 (39.7)
A lot	193 (50.2)
Missing	11 (2.9)
News or media reports	
Not at all	36 (9.4)
A little	181 (47.0)
Some	151 (39.2)
A lot	7 (1.8)
Missing	10 (2.6)
Professional society recommendations or guidelines	
Not at all	3 (0.8)
A little	9 (2.4)
Some	123 (31.9)
A lot	243 (63.1)
Missing	7 (1.8)
CDC reports	
Not at all	19 (4.9)
A little	49 (12.7)
Some	131 (34.0)
A lot	174 (45.3)
Missing	12 (3.1)
World Health Organization reports	
Not at all	20 (5.2)
A little	31 (8.1)
Some	144 (37.4)
A lot	179 (46.4)
Missing	11 (2.9)
Based on the results of your most recent search for information about COVID-19, how much do you agree or disagree with the following statements?	
It took a lot of effort to get the information I needed	
Strongly disagree	65 (16.9)
Somewhat disagree	155 (40.3)
Somewhat agree	135 (35.1)
Strongly agree	27 (7.0)
Missing	3 (0.7)
I felt frustrated during my search for the information	
Strongly disagree	61 (15.8)
Somewhat disagree	157 (40.8)
Somewhat agree	124 (32.2)
Strongly agree	38 (9.9)
Missing	5 (1.3)
I was concerned about the quality of the information	
Strongly disagree	30 (7.8)
Somewhat disagree	70 (18.2)
Somewhat agree	199 (51.7)
Strongly agree	82 (21.3)
Missing	4 (1.0)
The information I found was hard to understand	
Strongly disagree	145 (37.7)
Somewhat disagree	171 (44.4)
Somewhat agree	59 (15.3)
Strongly agree	5 (1.3)
Missing	5 (1.3)

a Percentages do not sum to 100. CDC = Centers for Disease Control and Prevention.

In a sample of oncology physicians practicing across the United States, we found a diverse array of reported sources for COVID-19 information that most commonly included professional societies, hospitals, and the CDC; however, trust in these sources of information varied widely, with professional societies the most trusted source. However, although we found that oncology physicians trust sources such as professional societies, they also report concerns about the quality of COVID-19–related information, which is consistent with early editorials and viewpoints published in the literature from leading health-care professionals (8,9). These individuals recognized early on in the pandemic a need to balance rapid publication of information on disease transmission, characteristics, and outcomes of individuals diagnosed with COVID-19 with rigorous reporting standards, extensive follow-up, and validation, which may translate into initial concerns with early published data on COVID-19 outcomes.

Initial reports suggest that COVID-19 may be particularly lethal in patients with cancer (10,11). These findings highlight sources of information most utilized by oncology physicians that can be targeted for up-to-date information on best practices around cancer care delivery, treatment modifications, and allocation of limited health-care resources during this crisis. Additionally, because professional societies are reported as one of the most trusted sources for COVID-19 information, these findings suggest that societies may consider added review to their posted information to ensure it is consistent with the continually evolving literature and of high scientific quality. Further, added efforts to ensure treatment standards and scientific evidence are easily accessible on these venues are warranted, because we report over than 40% of oncology physicians reported undertaking “a lot of effort” to identify COVID-19–related information.

Limitations of the study include reliance on convenience sampling to identify respondents, an inability to directly compare characteristics of nonrespondents, targeting of social media groups to reach a broad audience quickly, potential overrepresentation of female oncology physicians relative to the general oncology workforce, lack of detailed information on concerns with specific sources of COVID-19 information, and lower proportion of respondents from some states with the highest impact from COVID-19.

Despite these limitations, we provide current insights on COVID-19 information seeking from a large population of physicians currently treating cancer patients around the United States, highlighting the important role that professional societies, hospitals, and the CDC play in ensuring dissemination of high-quality practice recommendations for oncology physicians. These data provide an important starting point for understanding to best provide information to oncology physicians as the pandemic evolves as well as plan for dissemination of information in future outbreaks.

## Funding

This research was supported in part by the National Institutes of Health’s National Center for Advancing Translational Sciences grant UL1TR002494 as well as the National Cancer Institute P30 Cancer Center Support Grant CA77598.

## Notes

**Role of the funder:** The funders had no role in the design and conduct of the study; collection, management, analysis, and interpretation of the data; preparation, review, or approval of the manuscript; and decision to submit the manuscript for publication.

**Disclosures:** There are no financial disclosures or conflicts of interest to report.

**Disclaimer:** The content is solely the responsibility of the authors and does not necessarily represent the official views of the National Institutes of Health’s National Center for Advancing Translational Sciences or the National Cancer Institute.

**Author contributions:** HP, JH, RV: Conception and design. RV: Data acquisition and analysis. HP: Drafted initial manuscript. All authors: Drafting, revising, editing manuscript.

## Data Availability

Data available upon request from the corresponding author. 

## Supplementary Material

pkaa095_Supplementary_Data
