# Supplementary material for: Sources of Medical Information for Oncology Physicians During the COVID-19 Pandemic: Results From a National Cross-Sectional Survey
Source: JNCI Cancer Spectr. 2020 Oct 24;4(6):pkaa095. doi: 10.1093/jncics/pkaa095 (PMC7665641; doi:10.1093/jncics/pkaa095)
Supplement: pkaa095_Supplementary_Data [file pkaa095_supplementary_data.pdf]

COVID-19 and Oncologists Survey

INFORMATION SHEET FOR RESEARCH

COVID-19 and Oncologists Survey

You are invited to be in a study to understand how the COVID-19 pandemic is affecting the decision-making and emotional health of physicians who treated cancer patients in the United States. You were identified as a possible participant because you are a member of a national cancer oncology organization or you viewed the study on social media.

This study is being conducted by Rachel I. Vogel, PhD, University of Minnesota, Department of Obstetrics, Gynecology and Women's Health.

Procedures:

If you agree to be in this study, we would ask you to take an online, anonymous survey which will take you about 10 minutes to complete.

Confidentiality:

The records of this study will be kept private. In any sort of report we might publish, we will not include any information that will make it possible to identify a subject. Research records will be stored securely and only researchers will have access to the records.

Voluntary Nature of the Study:

Participation in this study is voluntary. Your decision whether or not to participate will not affect your current or future relations with the University of Minnesota. If you decide to participate, you are free to not answer any question or withdraw at any time without affecting those relationships.

Contacts and Questions:

The researcher conducting this study is: Rachel I. Vogel, Ph.D. If you have questions later, you are encouraged to contact them at the University of Minnesota, 420 Delaware Street SE MMC 395 Minneapolis, MN 55455, phone: 612-624-6928, email: umncancersurv@umn.edu.

This research has been reviewed and approved by an IRB within the Human Research Protections Program (HRPP). To share feedback privately with the HRPP about your research experience, call the Research Participants' Advocate Line at 612-625-1650 (Toll Free: 1-888-224-8636) or go to z.umn.edu/participants. You are encouraged to contact the HRPP if:

Your questions, concerns, or complaints are not being answered by the research team. You cannot reach the research team. You want to talk to someone besides the research team. You have questions about your rights as a research participant. You want to get information or provide input about this research. You can download a copy of this information to keep for your records.

[Attachment: "Information_Sheet_03252020.docx"]

Please confirm you are eligible for this study before proceeding:

You are a physician who offers cancer treatments to patients as a surgeon, medical oncologist, or radiation oncologist

◯ Yes
◯ No

You live in and are currently practicing in the United States

◯ Yes
◯ No

You can read and write in English

◯ Yes
◯ No

What is your biologic sex (sex/gender assigned at birth)?

◯ Male
◯ Female
◯ Intersex
◯ Prefer not to answer

Do you identify as transgender, gender-queer, gender-fluid, or another non-binary gender?

◯ Yes
◯ No
◯ I don't know
◯ Prefer not to answer

There are regional differences in the burden of COVID-19, and these differences are likely to change with time. Therefore, in order to understand your context better, we would like to know what state/territory you are currently residing/practicing in?

◯ Alabama (AL)
◯ Alaska (AK)
◯ Arizona (AZ)
◯ Arkansas (AR)
◯ California (CA)
◯ Colorado (CO)
◯ Connecticut (CT)
◯ Delaware (DE)
◯ District of Columbia (DC)
◯ Florida (FL)
◯ Georgia (GA)
◯ Hawaii (HI)
◯ Idaho (ID)
◯ Illinois (IL)
◯ Indiana (IN)
◯ Iowa (IA)
◯ Kansas (KS)
◯ Kentucky (KY)
◯ Louisiana (LA)
◯ Maine (ME)
◯ Maryland (MD)
◯ Massachusetts (MA)
◯ Michigan (MI)
◯ Minnesota (MN)
◯ Mississippi (MS)
◯ Missouri (MO)
◯ Montana (MT)
◯ Nebraska (NE)
◯ Nevada (NV)
◯ New Hampshire (NH)
◯ New Jersey (NJ)
◯ New Mexico (NM)
◯ New York (NY)
◯ North Carolina (NC)
◯ North Dakota (ND)
◯ Ohio (OH)
◯ Oklahoma (OK)
◯ Oregon (OR)
◯ Pennsylvania (PA)
◯ Rhode Island (RI)
◯ South Carolina (SC)
◯ South Dakota (SD)
◯ Tennessee (TN)
◯ Texas (TX)
◯ Utah (UT)
◯ Vermont (VT)
◯ Virginia (VA)
◯ Washington (WA)
◯ West Virginia (WV)
◯ Wisconsin (WI)
◯ Wyoming (WY)
◯ American Samoa (AS)
◯ Guam (GU)
◯ Northern Mariana Islands (MP)
◯ Puerto Rico (PR)
◯ Virgin Islands (VI)

Do you currently work in a clinical setting?

◯ Yes - Full Time
◯ Yes - Part Time
◯ No
◯ Retired

The following questions are specifically related to the current COVID-19 (coronavirus 2019) pandemic.

Are you concerned that some people do not understand the seriousness of COVID-19?

Not at all concerned — A great deal concerned

(Place a mark on the scale above)

In what size hospital or clinic do you currently practice?

◯ Small hospital (fewer than 100 beds)
◯ Medium hospital (100-499 beds)
◯ Large hospital (500 or more beds)
◯ Ambulatory clinic only (no inpatients)

Do you practice at an academic institution?

◯ Yes
◯ No

How much of your routine practice has been reduced as a result of COVID-19?

◯ None
◯ < 25% reduction
◯ 26-50% reduction
◯ 51-75% reduction
◯ >75% reduction
◯ Not applicable

How much personal financial burden will you incur as a result of COVID-19 and subsequent reduction in your "routine" patient volume?

None at all — A great deal

(Place a mark on the scale above)

Do you consider yourself to be at "high risk" for severe illness from COVID-19?

◯ Yes
◯ No
◯ Unsure

Indicate why you consider yourself to be at "high risk" for severe illness from COVID-19. Check all that apply.

☐ Older adult (>60 years old)
☐ History of cancer (completed active cancer treatment prior to January 2020)
☐ Immune-compromised (ex. HIV, bone marrow transplant, organ transplant, chemotherapy, taking immune-supressing drugs for Crohn's disease or rheumatoid arthritis)
☐ Heart disease
☐ Diabetes
☐ Lung disease (e.g. asthma, emphysema/COPD)
☐ Pregnant
☐ Active cancer treatment other than chemotherapy (recent surgery, recent/ongoing radiation therapy, recent/ongoing endocrine therapy)
☐ Obesity
☐ Active smoker
☐ Previous/Former smoker
☐ Other reason

Please specify

____

Have you been directly exposed to someone with COVID-19?

◯ Yes - at work
◯ Yes - outside of work (travel, community exposure, etc)
◯ No
◯ Unsure

How were you exposed to someone with COVID-19?

◯ COVID-19 positive patient
◯ COVID-19 positive healthcare worker
◯ COVID-19 positive staff who does not work face-to-face with patients
◯ Unsure

How were you exposed to someone with COVID-19?

◯ International travel
◯ Domestic travel (air, land)
◯ Community exposure
◯ Unsure

Do you have adequate PPE (Personal Protective Equipment) in your clinical practice?

◯ Yes
◯ No

Have you been diagnosed with COVID-19?

◯ Yes - confirmed with a positive test
◯ Yes - presumed
◯ No
◯ Unsure

How concerned are you about getting COVID-19?

◯ Not at all concerned
◯ Slightly concerned
◯ Somewhat concerned
◯ Moderately concerned
◯ Extremely concerned

How concerned are you about one of your family members getting COVID-19 from you?

◯ Not at all concerned
◯ Slightly concerned
◯ Somewhat concerned
◯ Moderately concerned
◯ Extremely concerned
◯ Already happened
◯ Not applicable

How concerned are you about your patients getting COVID-19 from you?

◯ Not at all concerned
◯ Slightly concerned
◯ Somewhat concerned
◯ Moderately concerned
◯ Extremely concerned
◯ Not applicable

To what degree has COVID-19 interfered with your ability to provide treatment to active cancer patients?

No problem | Severe problem

(Place a mark on the scale above)

How concerned are you about your patients getting the level of healthcare they need if they become extremely ill from something other than COVID-19?

- ◯ Not at all concerned
- ◯ Slightly concerned
- ◯ Somewhat concerned
- ◯ Moderately concerned
- ◯ Extremely concerned
- ◯ Not applicable

What is your medical specialty? Check all that apply.

- ☐ Surgeon
- ☐ Medical Oncology
- ☐ Radiation Oncology
- ☐ Gynecologic Oncology
- ☐ Other

Please specify.

____

Have you had to cancel/postpone your patients' cancer surgery?

- ◯ Yes
- ◯ No, but plan to continue to reassess this
- ◯ No

Are you referring patients for alternative treatment (chemotherapy, endocrine therapy, etc) in the meantime?

- ◯ Yes
- ◯ Sometimes
- ◯ No

What prompted you to cancel/postpone surgeries? Check all that apply

- ☐ Personal concern about my patients' exposure risk
- ☐ Personal concern about exposure risks to other hospitalized patients
- ☐ Personal concern about hospital staff and healthcare worker exposure risk
- ☐ Personal concern about blood bank shortages
- ☐ Desire to conserve PPE at my institution
- ☐ Divisional or departmental mandate due to COVID-19
- ☐ Institutional mandate due to COVID-19
- ☐ Surgeon general or professional medical organization recommendation (e.g. American College of Surgeons, etc)
- ☐ Closure of patient and family accommodations (such as American Cancer Society Hope Lodge)
- ☐ Strict visitor policy resulting in lack of family support for certain procedures
- ☐ Other

Please specify.

____

Have you had to alter your patients' chemotherapy plans?

◯ Yes
◯ No, but plan to continue to reassess this
◯ No

What prompted you to alter chemotherapy plans? Check all that apply.

☐ Personal concern about my patients' exposure risk
☐ Personal concern about exposure risks to other patients
☐ Personal concern about hospital/clinic staff and healthcare worker exposure risk
☐ Desire to conserve PPE at my institution
☐ Divisional or departmental mandate due to COVID-19
☐ Institutional mandate due to COVID-19
☐ Professional medical organization recommendation
☐ Closure of patient and family accommodations (such as American Cancer Society Hope Lodge)
☐ Strict visitor policy resulting in lack of family support for certain procedures
☐ Transportation concerns due to suspension of medical transportation services
☐ Other

Please specify.

____

Have you had to alter your patients' radiation plans?

◯ Yes
◯ No, but plan to continue to reassess this
◯ No

What prompted you to alter radiation plans? Check all that apply.

☐ Personal concern about my patients' exposure risk
☐ Personal concern about exposure risks to other patients
☐ Personal concern about hospital/clinic staff and healthcare worker exposure risk
☐ Desire to conserve PPE at my institution
☐ Divisional or departmental mandate due to COVID-19
☐ Institutional mandate due to COVID-19
☐ Professional medical organization recommendation
☐ Closure of patient and family accommodations (such as American Cancer Society Hope Lodge)
☐ Strict visitor policy resulting in lack of family support for certain procedures
☐ Transportation concerns due to suspension of medical transportation services
☐ Other

Please specify.

____

How do you decide which patients should receive an alternate treatment plan?

◯ Group consensus
◯ Physician choice
◯ Mutual decision of patient and physician
◯ Patient choice

What factors do you consider when deciding to change the treatment plan? 1=Most important to 9=Least important.

	1 - Most Important	2	3	4	5	6	7	8	9 - Least Important
Use of clinic/hospital resources	◯	◯	◯	◯	◯	◯	◯	◯	◯
Risk of patient exposure to coronavirus	◯	◯	◯	◯	◯	◯	◯	◯	◯
Risk of severe manifestation of COVID-19	◯	◯	◯	◯	◯	◯	◯	◯	◯
Presence of alternative treatment options	◯	◯	◯	◯	◯	◯	◯	◯	◯
Overall cancer prognosis/anticipated benefit of the treatment	◯	◯	◯	◯	◯	◯	◯	◯	◯
Anticipated rate of cancer growth/spread	◯	◯	◯	◯	◯	◯	◯	◯	◯
Patient proximity to the health system / patient and family accommodations	◯	◯	◯	◯	◯	◯	◯	◯	◯
Age of the patient	◯	◯	◯	◯	◯	◯	◯	◯	◯
Other patient comorbidities	◯	◯	◯	◯	◯	◯	◯	◯	◯

What has been the hardest part of your care of cancer patients during the COVID-19 pandemic?

These next questions ask about sources of health information and your level of trust of these sources.

Which of the following sources do you use for information about COVID-19? Check all that apply.

☐ Physician grand rounds/talks
☐ Hospital/Institution communications/emails
☐ Social media
☐ Social media - physician-only groups
☐ Literature search
☐ News/media reports
☐ Professional society recommendations/guidelines (e.g. ASCO, ASTRO, ACS, SSO, etc)
☐ CDC (Centers for Disease Control and Prevention) reports
☐ World Health Organization reports
☐ Other
☐ I have not looked for information about COVID-19

Please specify.

____

In general, how much would you trust information about health or medical topics from...

	Not at all	A little	Some	A lot
Physician grand rounds/talks	○	○	○	○
Hospital/Institution communications/emails	○	○	○	○
Social media	○	○	○	○
Social media - physician-only groups	○	○	○	○
Literature search	○	○	○	○
News/media reports	○	○	○	○
Professional society recommendations/guidelines (e.g. ASCO, ASTRO, ACS, SSO, etc)	○	○	○	○
CDC (Centers for Disease Control and Prevention) reports	○	○	○	○
World Health Organization reports	○	○	○	○

Based on the results of your most recent search for information about COVID-19, how much do you agree or disagree with the following statements?

	Strongly disagree	Somewhat disagree	Somewhat agree	Strongly agree
It took a lot of effort to get the information I needed.	○	○	○	○
I felt frustrated during my search for the information.	○	○	○	○
I was concerned about the quality of the information.	○	○	○	○
The information I found was hard to understand.	○	○	○	○

Over the last 2 weeks, how often have you been bothered by the following problems?

	Not at all	Several days	Over half the days	Nearly every day
Feeling nervous, anxious or on edge	◯	◯	◯	◯
Not being able to sleep or control worrying	◯	◯	◯	◯
Feeling down, depressed or hopeless	◯	◯	◯	◯
Little interest or pleasure in doing things	◯	◯	◯	◯

Finally, we just have a few demographic questions.

What is your current age?

____

What is your race? Select all that apply?

- ☐ White
- ☐ Black or African American
- ☐ American Indian or Alaska Native
- ☐ Asian Indian
- ☐ Chinese
- ☐ Filipino
- ☐ Japanese
- ☐ Korean
- ☐ Vietnamese
- ☐ Other Asian
- ☐ Native Hawaiian
- ☐ Guamanian or Chamorro
- ☐ Samoan
- ☐ Other Pacific Islander

Are you Hispanic, Latino/a, or of Spanish origin? Select all that apply.

- ☐ No, not of Hispanic, Lation/a, Spanish origin
- ☐ Yes, Mexican, Mexican American, Chicano/a
- ☐ Yes, Puerto Rican
- ☐ Yes, Cuban origin

Are you Hispanic, Latino/a, or of Spanish origin?

- ◯ Yes
- ◯ No

How many years have you been in practice?

____

What type(s) of cancer do you treat? Check all that apply.

☐ Bladder cancer
☐ Bone cancer
☐ Breast cancer
☐ Cervical cancer
☐ Colon cancer
☐ Endometrial/Uterine cancer
☐ Head/Neck cancer
☐ Hodgkins
☐ Renal cancer
☐ Leukemia
☐ Liver cancer
☐ Lung cancer
☐ Melanoma
☐ Non-Hodgkins
☐ Oral cancer
☐ Ovarian cancer
☐ Pancreatic cancer
☐ Pharyngeal cancer
☐ Prostate cancer
☐ Rectal cancer
☐ Sarcoma
☐ Skin cancer (not melanoma)
☐ Other

Please specify

____

Do you have children under the age of 18 living with you and/or under your care?

◯ Yes
◯ No

What type of community do you practice in?

◯ Rural area
◯ Small city or town
◯ Suburb near a large city
◯ Large city
